# Association between a four-parameter inflammatory index and all-cause mortality in critical ill patients with non-traumatic subarachnoid hemorrhage: a retrospective analysis of the MIMIC-IV database (2012-2019)

**DOI:** 10.3389/fimmu.2023.1235266

**Published:** 2023-10-23

**Authors:** Yong-Wei Huang, Ye Zhang, Zong-Ping Li, Xiao-Shuang Yin

**Affiliations:** ^1^ Department of Neurosurgery, Mianyang Central Hospital, School of Medicine, University of Electronic Science and Technology of China, Mianyang, Sichuan, China; ^2^ Department of Immunology, Mianyang Central Hospital, School of Medicine, University of Electronic Science and Technology of China, Mianyang, Sichuan, China

**Keywords:** pan-immune-inflammation value (PIV), subarachnoid hemorrhage (SAH), mortality, biomarkers, MIMIC-IV, propensity score matching (PSM)

## Abstract

**Background:**

Non-traumatic subarachnoid hemorrhage (SAH), primarily due to the rupture of intracranial aneurysms, contributes significantly to the global stroke population. A novel biomarker, pan-immune-inflammation value (PIV) or called the aggregate index of systemic inflammation (AISI), linked to progression-free survival and overall survival in non-small-cell lung cancer and mortality in Coronavirus Disease 2019 (COVID-19) patients, has surfaced recently. Its role in non-traumatic SAH patients, however, remains under-researched. This study aims to determine the relationship between PIV and all-cause mortality in non-traumatic SAH patients.

**Methods:**

A retrospective analysis was conducted using data from the Medical Information Mart for Intensive Care (MIMIC-IV) database to examine the association between PIV and all-cause mortality in critically ill patients with non-traumatic SAH. PIV measurements were collected at Intensive Care Unit (ICU) admission, and several mortality measures were examined. To control for potential confounding effects, a 1:1 propensity score matching (PSM) method was applied. The optimal PIV cutoff value was identified as 1362.45 using X-tile software that is often used to calculate the optimal cut-off values in survival analysis and continuous data of medical or epidemiological research. The relationship between PIV and short- and long-term all-cause mortality was analyzed using a multivariate Cox proportional hazard regression model and Kaplan-Meier (K-M) survival curve analysis. Interaction and subgroup analyses were also carried out.

**Results:**

The study included 774 non-traumatic SAH patients. After PSM, 241 pairs of score-matched patients were generated. The Cox proportional hazard model, adjusted for potential confounders, found a high PIV (≥ 1362.45) independently associated with 90-day all-cause mortality both pre- (hazard ratio [HR]: 1.67; 95% confidence intervals (CI): 1.05-2.65; P = 0.030) and post-PSM (HR: 1.58; 95% CI: 1.14-2.67; P = 0.042). K-M survival curves revealed lower 90-day survival rates in patients with PIV ≥ 1362.45 before (31.1% vs. 16.1%%, P < 0.001) and after PSM (68.9% vs. 80.9%, P < 0.001). Similarly, elevated PIV were associated with increased risk of ICU (pre-PSM: HR: 2.10; 95% CI: 1.12-3.95; P = 0.02; post-PSM: HR: 2.33; 95% CI: 1.11-4.91; P = 0.016), in-hospital (pre-PSM: HR: 1.91; 95% CI: 1.12-3.26; P = 0.018; post-PSM: 2.06; 95% CI: 1.10-3.84; P = 0.034), 30-day (pre-PSM: HR: 1.69; 95% CI: 1.01-2.82; P = 0.045; post-PSM: 1.66; 95% CI: 1.11-2.97; P = 0.047), and 1-year (pre-PSM: HR: 1.58; 95% CI: 1.04-2.40; P = 0.032; post-PSM: 1.56; 95% CI: 1.10-2.53; P = 0.044) all-cause mortality. The K-M survival curves confirmed lower survival rates in patients with higher PIV both pre- and post PSM for ICU (pre-PSM: 18.3% vs. 8.4%, P < 0.001; post-PSM:81.7 vs. 91.3%, P < 0.001), in-hospital (pre-PSM: 25.3% vs. 12.8%, P < 0.001; post-PSM: 75.1 vs. 88.0%, P < 0.001), 30-day (pre-PSM: 24.9% vs. 11.4%, P < 0.001; post-PSM:74.7 vs. 86.3%, P < 0.001), and 1-year (pre-PSM: 36.9% vs. 20.8%, P < 0.001; P = 0.02; post-PSM: 63.1 vs. 75.1%, P < 0.001) all-cause mortality. Stratified analyses indicated that the relationship between PIV and all-cause mortality varied across different subgroups.

**Conclusion:**

In critically ill patients suffering from non-traumatic SAH, an elevated PIV upon admission correlated with a rise in all-cause mortality at various stages, including ICU, in-hospital, the 30-day, 90-day, and 1-year mortality, solidifying its position as an independent mortality risk determinant. This study represents an attempt to bridge the current knowledge gap and to provide a more nuanced understanding of the role of inflammation-based biomarkers in non-traumatic SAH. Nevertheless, to endorse the predictive value of PIV for prognosticating outcomes in non-traumatic SAH patients, additional prospective case-control studies are deemed necessary.

## Introduction

Non-traumatic subarachnoid hemorrhage (SAH) represents a critical medical condition, primarily attributable to the rupture of intracranial aneurysms, contributing to 2–7% of overall stroke cases (1). The disease burden correlated with non-traumatic SAH is indeed profound and perhaps underappreciated. Remarkably, half of the non-traumatic SAH incidence arises in individuals aged below 60, with approximately one-third of these patients losing their lives before hospital admission. Consequently, immediate interventions in the intensive care unit (ICU) become indispensable for the remaining patients (1, 2). Regrettably, even with the execution of the best management protocols in the ICU, the in-hospital mortality rates associated with non-traumatic SAH remain alarmingly high (3). Consistent epidemiological studies have manifested the escalated prevalence of non-traumatic SAH, registering in-hospital mortality rates as high as 40% (1). Considering the potentially fatal implications of non-traumatic SAH, there is a pressing requirement for cost-effective, non-invasive diagnostic tools that can identify individuals at an elevated risk of mortality, thereby enabling timely implementation of preventative measures to reduce the likelihood of fatal consequences.

Systemic inflammation is hypothesized to play a significant role in the onset and progression of non-traumatic subarachnoid hemorrhage (SAH) due to its common risk factors and inflammatory profiles shared with certain inflammatory diseases (4, 5). The exact pathophysiological pathways through which inflammation catalyzes non-traumatic SAH’s evolution are yet to be comprehensively elucidated; however, they are postulated to encompass leukocytosis and platelet aggregation (6–8). It has been observed that elevated platelet aggregation and systemic inflammation are directly related to early brain injury and contribute to non-traumatic SAH’s pathogenesis. Additionally, inflammatory and thrombotic processes have been identified as crucial players in the underlying pathophysiological mechanism (7, 9), thereby amplifying the predisposition to delayed cerebral ischemia (DCI) subsequent to non-traumatic SAH.

The potential of systemic inflammatory indices derived from complete blood count (CBC) tests, such as the neutrophil/lymphocyte ratio (NLR), derived-NLR, platelet/lymphocyte ratio (PLR), and monocyte/lymphocyte ratio (MLR), has recently been highlighted. These indices, with their cost-effective and accessible nature, demonstrate considerable predictive utility across a variety of disorders, including non-traumatic SAH (10–16). Further, the systemic inflammation response index (SIRI) and systemic immune-inflammation index (SII) offer innovative, comprehensive biomarkers derived from three distinct blood cell counts. SIRI employs the absolute values of peripheral neutrophil, monocyte, and lymphocyte counts (N×M/L) (17), while SII uses counts of platelets, neutrophils, and lymphocytes (N×P/L) (18). Both SIRI and SII have shown valuable predictive capacity regarding clinical outcomes and severity in SAH patients (19–23). The pan-immune-inflammation value (PIV), also called the aggregate index of systemic inflammation (AISI), is computed by multiplying the counts of neutrophils, monocytes, and platelets, followed by dividing the result by the lymphocyte count (N×M×P/L). These four types of blood cells reflect different inflammatory and immune pathways in the body and can provide a more comprehensive reflection of the body’s inflammatory status. PIV was widely applied in studies of cancer that two meta-analyses have demonstrated that PIV is a prognostic biomarker of overall survival and progression-free survival in cancer patients (24, 25), while AISI is usually used in studies of COVID-19 (26–28) as well as malignant conditions, including non-small-cell lung cancer (29), esophageal cancer (30), and prostate cancer (31). In fact, these two indices are same but with different names and they shared the same formula. The cells incorporated in this four-parameter inflammatory index calculation are crucial for maintaining equilibrium in the immune system, which safeguards the body against pathogens and diseases. Yet, these cells can also generate pro-inflammatory substances linked with various inflammatory diseases (32, 33).

Considering the widespread utilization of PIV and the heavy burden of stroke globally, we hypothesize that PIV has a similar predictive ability for mortality in non-traumatic SAH patients as that in COVID-19 and caner patients. Consequently, this study was designed with the aim of identifying the relationship between PIV and all-cause mortality in patients suffering from non-traumatic SAH. Besides, we hope the findings will provide new insight into how to manage non-traumatic SAH patients for clinicians.

## Methods

### Data source

This study employed a retrospective cohort design, utilizing the Medical Information Mart for Intensive Care (MIMIC-IV) database (version 2.2) (34). MIMIC-IV, a publicly accessible critical care database, is renowned for its extensive clinical data on patients treated in intensive care units. Acknowledged as one of the most voluminous and frequently engaged databases in intensive care medicine, it offers crucial resources for research and analytic purposes. With a wide spectrum of ICU-related data, MIMIC-IV serves as an invaluable tool for investigating critical care outcomes, predictive modeling, clinical decision support, and additional research areas. Permission to use the MIMIC-IV database in this study was obtained from the Massachusetts Institute of Technology and the Institutional Review Board of Beth Israel Deaconess Medical Center (BIDMC, Boston, MA, USA).

### Ethical considerations and data privacy

To ensure ethical standards and patient privacy, the data used in this study underwent de-identification, with all precautions taken to uphold patient confidentiality. The author, Yong-Wei Huang, successfully completed the “Protecting Human Research Participants” web-based course offered by the National Institutes of Health (Record ID: 12150448) and was thereby authorized to access the MIMIC-IV database for data extraction. Given the de-identified nature of the data, the Beth Israel Deaconess Medical Center’s ethical committee waived the requirement for informed consent.

### Study population, variable extraction, and outcomes

**Figure 1:**
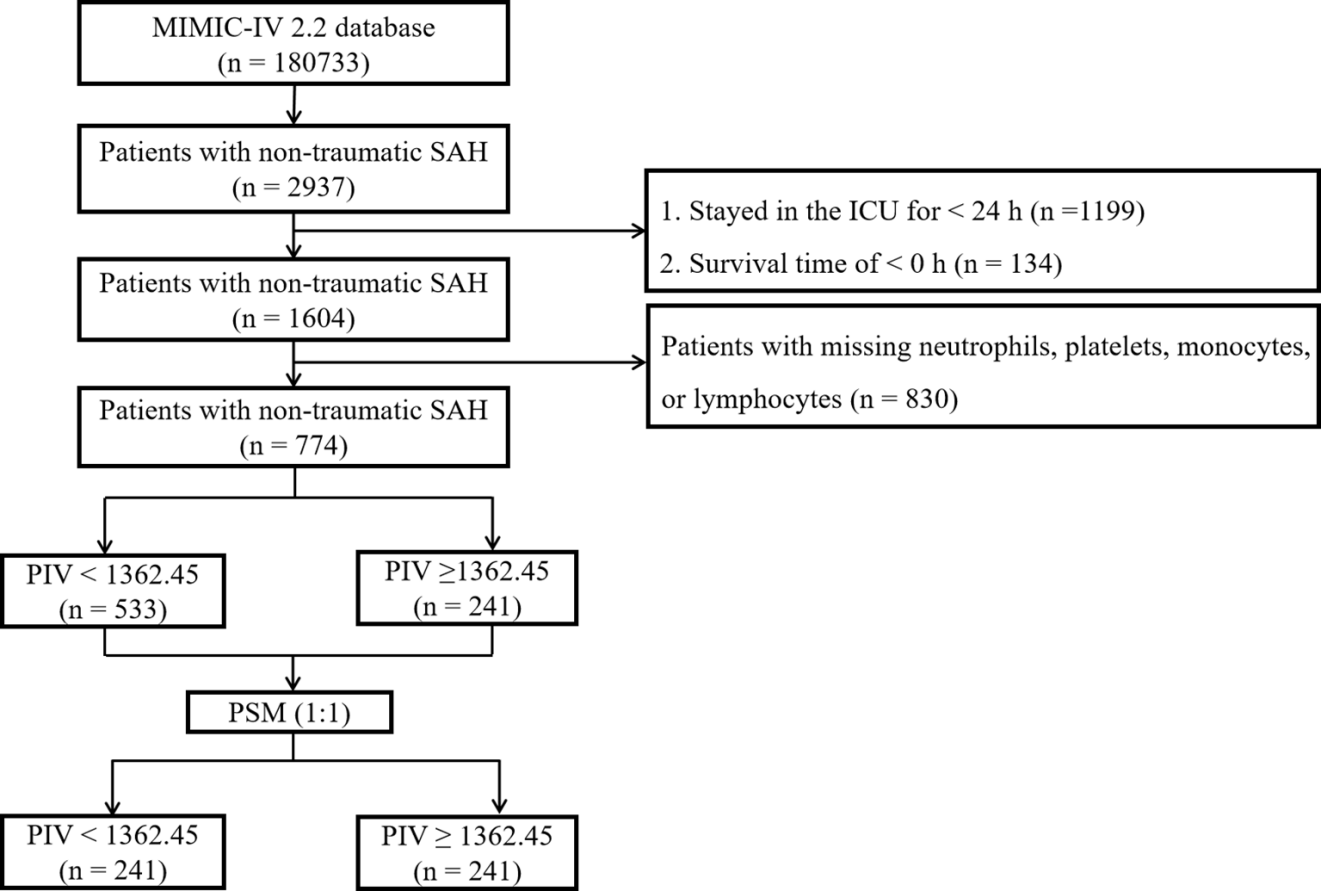
A flowchart for the patient selection process.

The MIMIC-IV database contained data for a total of 180,733 individuals admitted to the ICU between 2012 and 2019. Out of these, 2,937 patients were identified as having non-traumatic subarachnoid hemorrhage (SAH) based on ICD-9 and ICD-10 codes (ICD-9 code 430 and ICD-10 codes I60, I600 to I6012, I6000 to I6002, I6020 to I6022, I6030 to I6032, and I6050 to I6052). For this investigation, only patients aged 18 years and above were initially considered, and data from their initial ICU stay were gathered. Exclusion criteria encompassed patients with missing values for neutrophils, platelets, monocytes, or lymphocytes post ICU admission, those with an ICU stay duration of less than 24 hours, and those with a negative survival time. Subsequently, a total of 774 patients met the inclusion standards and were incorporated in the final analysis (**Figure 1**).

The primary variable of interest in this study was the first blood routine obtained post admission to the ICU, which was viewed as the primary exposure factor. All variables used were extracted from the MIMIC-IV database using Structured Query Language (SQL) with PostgreSQL. The extraction process covered five main components: demographic variables, clinical severity upon admission, vital signs, comorbidities, and laboratory variables. To handle any missing data, the predicted mean matching method was utilized to impute values in the dataset. There were 124 missing values in the matching variables (**Supplemental Table 1**). The missing values in the matching variables occurred primarily due to missing completely at random. We conducted a comparison between the results obtained using the predicted mean matching method for imputing missing values and the results from a complete case analysis. The results were completely consistent. The study’s primary endpoint was 90-day mortality, while secondary endpoints comprised ICU mortality, in-hospital mortality, 30-day mortality, and 1-year all-cause mortality. Importantly, the 90-day mortality was defined as the death within 90 days after SAH rather than a simple dead status at the 90^th^ days from the ICU admission. The definitions of secondary endpoints are similar to this.

For an exhaustive list of the extracted variables, refer to **Table 1**.

**Table 1:** The detailed extracted variables in MIMIC-IV database.

Items	Composition
Demographic variables	Age, sex, ethnicity
Clinical severity on admission	GCS, SAPS II
Vital signs	SBP, DBP, MBP, temperature, heart rate, respiratory rate, SpO_2_
Comorbidities	Hypertension, diabetes mellitus, CHF, COPD, sepsis, RF, liver diseases, malignancy
Laboratory variables	WBC count, neutrophils count, monocytes count, lymphocytes count, platelets count, hemoglobin, glucose, chloride, creatinine, BUN
Treatments	Vasopressor, ventilation, oxygen
Clinical outcomes	ICU, in-hospital, 30-day, 90-day, and 1-year all-cause mortality

GCS, Glasgow Coma Scale; SAPS II, Simplified Acute Physiology Score; SBP, systolic blood pressure; DBP, diastolic blood pressure; MBP, mean blood pressure; SpO2, percutaneous oxygen saturation; CHF, congestive heart failure; COPD, chronic pulmonary disease; RF, renal failure; WBC, white blood cell; BUN, blood urea nitrogen; ICU, intensive care unit.

### Propensity score matching

Due to the inherent limitations of the retrospective study design, the patient selection process might have introduced selection bias and potential confounding factors. To mitigate these issues, we conducted a propensity score matching (PSM) analysis, which aimed to minimize the impact of bias and confounders. The PSM analysis involved creating a logistic regression model to compute propensity scores, subsequently utilized to match patients based on several variables. These variables, incorporated for calculating propensity scores, encompassed age, sex, ethnicity, GCS, SAPS II, SBP, DBP, MBP, temperature, heart rate, RR, SpO2, Hypertension, diabetes mellitus, CHF, COPD, sepsis, RF, liver diseases, malignancy, WBC count, hemoglobin, glucose, chloride, creatinine, BUN, vasopressor, ventilation, and oxygen. The PSM analysis employed a 1:1 nearest neighbor matching algorithm with a caliper of 0.1. To evaluate the balance between the two groups, we calculated absolute standardized differences (ASDs) pre and post-matching. ASDs below 0.10 implied a well-balanced distribution of characteristics between the matched groups.

### Statistical analysis

Continuous variables were presented as median with interquartile range (IQR), and their differences were analyzed using the t-test or Mann-Whitney U-test. Categorical variables were reported as counts with proportions and compared using the Chi-square test or Fisher’s exact test. To determine the optimal cutoff value for the PIV in relation to 90-day mortality, we employed X-tile software (Version 3.6.1, Yale University School of Medicine) that is often used to calculate the optimal cut-off values in survival analysis and continuous data of medical or epidemiological research. Consequently, PIV was dichotomized into two groups using the pre-determined optimal cutoff value. Further, the choice of optimal cut-off point that maximized the risk ratio can be found in **Supplemental Figure 1**, as well as the relationship of PIV ≥1362.45 and the distribution of PIV. We assessed the proportional hazards assumption (PHA) using both graphical methods and statistical tests. Graphically, we employed Kaplan-Meier (K-M) curves to visualize the stability of hazard ratios (HRs) in survival analysis. For statistical tests, we performed the Schoenfeld residuals test and the Grambsch-Therneau test to formally assess the PHA assumption. In our study, we had censored data, meaning that some patients did not experience the event of interest by the end of the observation period. To handle censored data, we followed the standard practice in Cox regression by treating censored individuals as having experienced no events during the observation period. The time-to-event variable in our analysis was the time from ICU admission until the occurrence of the death at endpoint time of interest. Univariate and multivariate analyses of prognostic factors were performed using the Cox proportional hazards model to identify independent predictors of 90-day, ICU, in-hospital, 30-day, and 1-year mortality following non-traumatic SAH. The results were reported as HRs with 95% confidence intervals (CIs). We conducted subgroup analyses to investigate the impact of PIV on mortality within different subgroups. Stratification was carried out using a Cox regression model based on age (< 70 and ≥ 70 years), gender, hypertension, diabetes mellitus, liver disease, malignancy, and RF. Additionally, PIV was divided into four equal-interval quartiles to explore the relationship between varying PIV levels and all-cause mortality, with the first quartile serving as the reference group. To explore non-linear relationships, we employed restricted cubic splines (RCS). Smooth curve fitting and generalized additive models were used to investigate the threshold effect of PIV on the all-cause mortality in critical ill patients with non-traumatic SAH and identify the inflection point. All statistical analyses were two-sided, and p-values less than 0.05 were deemed statistically significant. The software used for analyses included R statistical software (R version 4.2.2, R Foundation for Statistical Computing), SPSS Statistics 26 (IBM, Chicago, IL, USA), and GraphPad Prism 8 (GraphPad Software, San Diego, CA, USA).

## Results

### Baseline characteristics of subjects

The study included a total of 774 out of 2937 non-traumatic SAH patients who received ICU treatment. There were 401 (51.8%) males and 373 (48.2%) females. The median age of the entire cohort was 62 (IQR, 51–76). Patients were divided into two groups based on the PIV optimal cutoff value determined by X-tile software, resulting in a low PIV group (< 1362.45) and a high PIV group (≥ 1362.45). Before propensity score matching, patients with higher PIV were found to have a higher SAPS II score, heart rate, and respiratory rate, as well as higher rates of sepsis, RF, ventilation, and vasopressor, elevated levels of WBC count, neutrophils, monocytes, platelets, glucose, BUN, and short- and long-term all-cause mortality. Further described, patients with PIV ≥ 1362.45 had a higher risk of various adverse outcomes compared to those in the PIV < 1362.45 group. They experienced a higher rates of ICU mortality (18.3% vs. 8.4%%, P < 0.001), in-hospital mortality (24.9% vs. 11.4%%, P < 0.001), 30-day mortality (25.3% vs. 12.8%, P < 0.001), 90-day mortality (31.1% vs. 16.1%%, P < 0.001), and 1-year mortality (36.9% vs. 20.8%%, P < 0.001). Nevertheless, no significant differences were observed between high PIV group and low PIV group in LOS ICU (P = 0.174) and LOS hospital (P = 0.052). More detailed results can be found in **Table 2**. It is worth noting that there was a rather high number of patients with missing inflammation markers, which were excluded from analyses. In order to further assess the possible bias introduced by excluding these patients, we analyzed the baseline characteristics of included individuals and excluded individuals, and most of the baseline characteristics between them were of no difference. Hence, we put the related statistical results in **Supplemental Table 2**.

**Table 2:** Baseline characteristics before propensity score matching.

Variable	Total (n=774)	PIV		*P* value
		< 1362.45 (n = 533)	≥ 1362.45 (n = 241)	
Demographics				
Age, years	62 (51-76)	61 (51-75)	66 (51-78)	0.158
Men, n (%)	401 (51.8%)	266 (49.9%)	135 (56.0%)	0.115
Ethnicity, n (%)				0.007
Asian	27 (3.5%)	14 (2.6%)	13 (5.4%)	
White	450 (58.1%)	310 (58.2%)	140 (58.1%)	
Black	45 (5.8%)	40 (7.5%)	5 (2.1%)	
Others	252 (32.6%)	169 (31.7%)	83 (34.4%)	
Clinical severity				
GCS	15 (14-15)	15 (14-15)	15 (14-15)	0.146
SAPS II	31 (24-39)	30 (24-37)	33 (25-41)	0.002
Vital signs				
SBP, mm Hg	132 (115-146)	132 (115-146)	132 (117-144)	0.912
DBP, mm Hg	72 (62-83)	72 (62-82)	72 (61-84)	0.806
MBP, mm Hg	88 (78-100)	88 (78-100)	88 (79-100)	0.833
Temperature	36.9 (36.6-37.2)	36.8 (36.6-37.1)	36.9 (36.6-37.2)	0.882
Heart rate	82 (71-94)	81 (70-93)	85 (73-98)	0.027
Respiratory rate	18 (15-21)	18 (15-21)	19 (16-22)	0.046
SpO_2_	99 (96-100)	98 (96-100)	99 (96-100)	0.092
Comorbidities				
Hypertension, n (%)	380 (49.1%)	255 (47.8%)	125 (51.9%)	0.300
Diabetes mellitus, n (%)	146 (18.9%)	100 (18.8%)	46 (19.1%)	0.915
CHF, n (%)	9 (1.2%)	8 (1.5%)	1 (0.4%)	0.287
COPD, n (%)	59 (7.6%)	37 (6.9%)	22 (9.1%)	0.288
Sepsis, n (%)	367 (47.4%)	224 (42.0%)	143 (59.3%)	<0.001
Malignancy, n (%)	92 (11.9%)	64 (12.0%)	28 (11.6%)	0.877
RF, n (%)	437 (56.5%)	287 (53.9%)	150 (62.2%)	0.029
Liver disease n (%)	98 (12.7%)	68 (12.8%)	30 (12.5%)	0.904
Laboratory parameters				
WBC counts, 10^9^/L	11 (8.3-14)	10.1 (7.5-13.1)	13.2 (10.3-16)	< 0.001
Neutrophils counts, 10^9^/L	7.7 (4.8-11.5)	5.8 (4-8.3)	12.7 (10.5-15.7)	< 0.001
Lymphocytes counts, 10^9^/L	1.4 (0.9-2)	1.6 (1-2.2)	1 (0.7-1.4)	< 0.001
Monocytes counts, 10^9^/L	0.7 (0.5-0.9)	0.6 (0.4-0.8)	1 (0.7-1.3)	< 0.001
Platelets counts, 10^9^/L	195 (159-240)	187 (153-225)	219 (175-268)	< 0.001
Hemoglobin (g/L)	11.9 (10.6-13.1)	11.9 (10.6-13)	11.9 (10.7-13.2)	0.315
Glucose (mmol/L)	127 (106-151)	123 (104-147)	134 (114-161)	< 0.001
Chloride	104 (101-107)	104 (101-107)	104 (100-107)	0.305
Creatinine	0.8 (0.7-1)	0.8 (0.6-1)	0.8 (0.7-1)	0.077
BUN	14 (10-20)	14(10-19)	15 (11-21)	0.005
PIV	722.4 (292.8-1626.8)	403.3 (200.3-758.9)	2366.8 (1728.4-3455.5)	< 0.001
Treatment				
Ventilation, n (%)	531 (68.6%)	346 (64.9%)	185 (76.7%)	0.001
Oxygen, n (%)	474 (61.2%)	330 (61.9%)	144 (59.8%)	0.567
Vasopressor, n (%)	197 (25.5%)	116 (21.8%)	81 (33.6%)	< 0.001
Clinical Outcomes				
LOS ICU, day	4 (2-10)	4 (2-9)	4 (1-13)	0.174
LOS Hospital, day	9 (4-18)	9 (5-16)	11 (4-22)	0.052
In-hospital mortality, n (%)	121 (15.6%)	61 (11.4%)	60 (24.9%)	< 0.001
ICU mortality, n (%)	89 (11.5%)	45 (8.4%)	44 (18.3%)	< 0.001
30-day mortality, n (%)	129 (16.7%)	68 (12.8%)	61 (25.3%)	< 0.001
90-day mortality, n (%)	161 (20.8%)	86 (16.1%)	75 (31.1%)	< 0.001
1-year mortality, n (%)	200 (25.8%)	111 (20.8%)	89 (36.9%)	< 0.001

PIV, pan-immune-inflammation value; GCS, Glasgow Coma Scale; SAPS II, Simplified Acute Physiology Score; SBP, systolic blood pressure; DBP, diastolic blood pressure; MBP, mean blood pressure; SpO2, percutaneous oxygen saturation; CHF, congestive heart failure; COPD, chronic pulmonary disease; RF, renal failure; WBC, white blood cell; BUN, blood urea nitrogen; LOS ICU, Length of ICU stay; LOS hospital, Length of hospital stay.

### Univariate and multivariate Cox regression models of PIV with mortality in patients with non-traumatic SAH before propensity score matching

To elucidate the potential relationship between the PIV and mortality outcomes in patients suffering from non-traumatic SAH, both univariate and multivariate Cox regression models were executed, with PIV categorized in binary. In the initial model without adjustments, a heightened PIV (≥ 1362.45) was demonstrably linked with escalated risks of mortality at various intervals: 90-day (HR = 2.07, 95% CI: 1.52-2.82, P < 0.001), within the ICU (HR = 1.95, 95% CI: 1.29-2.97, P = 0.002), during hospitalization (HR = 1.91, 95% CI: 1.34-2.73, P < 0.001), 30-day (HR = 2.16, 95% CI: 1.53-3.04, P < 0.001), and at the 1-year (HR = 1.97, 95% CI: 1.49-2.60, P < 0.001) for all causes of mortality. When adjusting for confounding variables such as age, gender, and ethnicity in multivariate model 1, the patient group exhibiting a PIV ≥ 1362.45 persisted in showing an elevated risk for mortality at the previously mentioned time points. An additional multivariate model (model 2), which incorporated further potential confounders (P < 0.05) identified in **Table 2**, further identified that a heightened PIV was independently linked with increased mortality risk across the aforementioned time intervals. Detailed data are presented in **Table 3**.

**Table 3:** Univariate and multivariate Cox regression models of PIV with mortality in patients with non-traumatic SAH before propensity score matching.

Outcome	Unadjusted		Model 1		Model 2	
	HR, 95% CI	P value	HR, 95% CI	P value	HR, 95% CI	P value
ICU mortality						
PIV (< 1362.45)	1 (Ref)		1 (Ref)		1 (Ref)	
PIV (≥1362.45)	1.95 (1.29-2.97)	0.002	1.62 (1.05-2.50)	0.029	2.10 (1.12-3.95)	0.021
In-hospital mortality						
PIV (< 1362.45)	1 (Ref)		1 (Ref)		1 (Ref)	
PIV (≥ 1362.45)	1.91(1.34-2.73)	< 0.001	1.57 (1.08-2.29)	0.018	1.91(1.12-3.26)	0.018
30-day mortality						
PIV (< 1362.45)	1 (Ref)		1 (Ref)		1 (Ref)	
PIV (≥ 1362.45)	2.16 (1.53-3.04)	< 0.001	1.86 (1.30-2.67)	<0.001	1.69 (1.01-2.82)	0.045
90-day mortality						
PIV (< 1362.45)	1 (Ref)		1 (Ref)		1 (Ref)	
PIV (≥ 1362.45)	2.07 (1.52-2.82)	< 0.001	1.84 (1.33-2.54)	< 0.001	1.67 (1.05-2.65)	0.030
1-year mortality						
PIV (< 1362.45)	1 (Ref)		1 (Ref)		1 (Ref)	
PIV (≥ 1362.45)	1.97 (1.49-2.60)	<0.001	1.74 (1.30-2.32)	< 0.001	1.58 (1.04-2.40)	0.032

Model 1: Unadjusted.

Model 2: Adjusted age, gender, and ethnicity.

Model 3: Adjusted all variables of P < 0.05 in **Table 2**.

**Figure 2:**
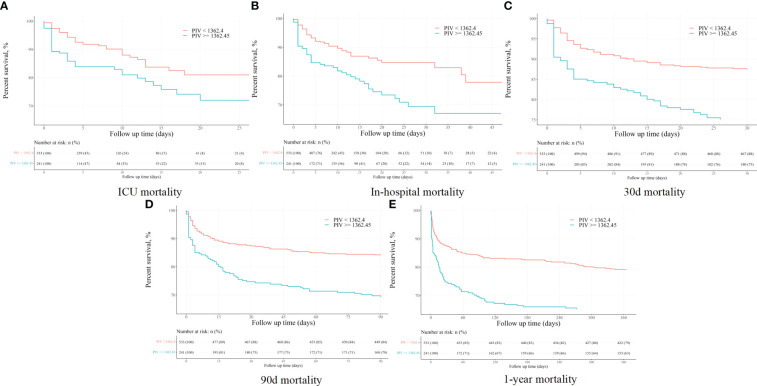
The K-M survival plots of **(A)** ICU, **(B)** in-hospital, **(C)** 30-day, **(D)** 90-day, and **(E)** 1-year mortality before propensity score matching.

The Kaplan-Meier (K-M) survival curves distinctly illustrated higher mortality rates at 90-day, ICU, in-hospital, 30-day, and 1-year intervals in patients with a PIV value ≥ 1362.45, as opposed to those with PIV < 1362.45 (31.1% vs. 16.1%, P < 0.001; 18.3% vs. 8.4%, P < 0.001; 25.3% vs. 12.8%, P < 0.001; 24.9% vs. 11.4%, P < 0.001; 36.9% vs. 20.8%, P < 0.001). More results can be found in **Figure 2**.

### Relationship between the PIV and all-cause mortality in non-traumatic SAH patients after propensity score matching

**Figure 3:**
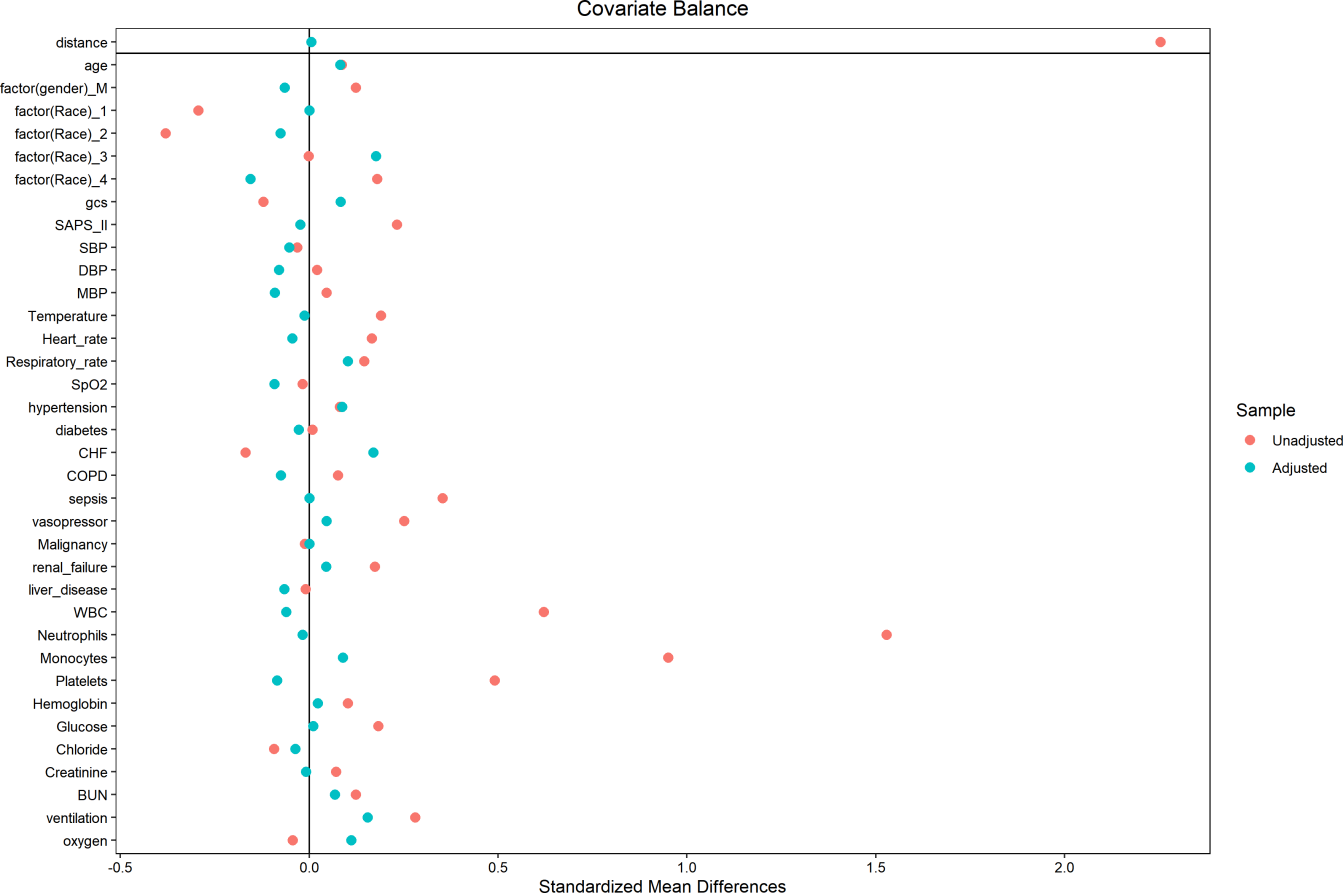
The absolute standardized differences for variables used to match the two groups.

In an effort to normalize the disparity in baseline features between the patient groups with low and high PIV, a 1:1 PSM analysis was executed, leading to the formation of 241 matched patient pairs. The demographics, comorbidities, majority of laboratory parameters, metrics, and treatments exhibited balance between the two cohorts post-PSM, as delineated in **Table 4**. However, elements such as neutrophil count, monocyte count, platelet count, and lymphocyte count that directly contribute to the PIV were not considered as matching variables. The efficacy of the matching process was evaluated by determining the absolute standardized differences (ASD) both prior to and following PSM, as illustrated in **Figure 3**.

**Table 4:** Baseline characteristics after propensity score matching.

Variable	Total (n=482)	PIV		*P* value
		< 1362.45 (n = 241)	≥ 1362.45 (n = 241)	
Demographics				
Age, years	66 (52-78)	65 (52-78)	66 (51-78)	0.434
Men, n (%)	265 (55.0%)	130 (53.9%)	135 (56.0%)	0.647
Ethnicity, n (%)				0.028
Asian	12 (2.5%)	9 (3.7%)	3 (1.2%)	
White	18 (3.7%)	13 (5.4%)	5 (2.1%)	
Black	286 (59.3%)	146 (60.6%)	140 (58.1%)	
Others	166 (34.4%)	73 (30.3%)	93 (38.6%)	
Clinical severity				
GCS	15 (14-15)	15 (14-15)	15 (14-15)	0.326
SAPS II	32.5 (25-40)	32 (26-39)	33 (25-41)	0.152
Vital signs				
SBP, mmHg	132 (117-146)	132 (116-147)	132 (117-144)	0.915
DBP, mmHg	72 (62-83)	71 (62-83)	72 (61-84)	0.383
MBP, mmHg	88 (78-100)	89 (77-100)	88 (79-100)	0.511
Temperature (°C)	36.9 (36.6-37.2)	36.9 (36.6-37.1)	36.9 (36.6-37.2)	0.063
Heart rate	83 (72-95)	81(71-93)	85 (73-98)	0.091
Respiratory rate	18 (15-21)	18 (15-21)	19 (16-22)	0.041
SpO_2_	99 (96-100)	98 (96-100)	99 (96-100)	0.003
Comorbidities				
Hypertension, n (%)	235 (48.8%)	110 (45.6%)	125 (51.9%)	0.172
Diabetes mellitus, n (%)	99 (20.5%)	53 (22.0%)	46 (19.1%)	0.430
CHF, n (%)	4 (0.8%)	3 (1.2%)	1 (0.4%)	0.315
COPD, n (%)	47 (9.8%)	25 (10.4%)	22 (9.1%)	0.645
Sepsis, n (%)	252 (52.3%)	109 (45.2%)	143 (59.3%)	0.002
Malignancy, n (%)	59 (12.2%)	31 (12.9%)	28 (11.6%)	0.097
Renal failure, n (%)	292 (60.6%)	142 (58.9%)	150 (62.2%)	0.455
Liver disease n (%)	56 (11.6%)	26 (10.8%)	30 (12.5%)	0.569
Laboratory parameters				
WBC counts, 10^9^/L	11.6 (9.2-14.3)	10.1 (8-12.8)	13.2 (10.3-16)	<0.001
Neutrophils counts, 10^9^/L	10.4 (7.7-13.4)	8.1 (6-10.2)	12.7 (10.5-15.7)	<0.001
Lymphocytes counts, 10^9^/L	1.1 (0.8-1.6)	1.2 (0.8-1.6)	1 (0.7-1.4)	0.017
Monocytes counts, 10^9^/L	0.8 (0.6-1.1)	0.7 (0.5-0.8)	1 (0.7-1.3)	<0.001
Platelets counts, 10^9^/L	203 (165-254)	192 (161-240)	219 (175-268)	<0.001
Hemoglobin (g/L)	11.9 (10.7-13.2)	11.9 (10.7-13.1)	11.9 (10.7-13.2)	0.619
Glucose (mmol/L)	129 (109-155)	125 (105-148)	134 (114-161)	<0.001
Chloride	104 (100-107)	104 (100-106)	104 (100-107)	0.793
Creatinine	0.8 (0.7-1)	0.8 (0.7-1)	0.8 (0.7-1)	0.922
BUN	15 (11-21)	14 (10-21)	15 (11-21)	0.351
PIV	1362.5 (789-2366.8)	789 (581-1018.3)	2366.8 (1728.4-3455.5)	<0.001
Treatment				
Ventilation, n (%)	344 (71.4%)	159 (66.0%)	185 (76.8%)	0.009
Oxygen, n (%)	295 (61.2%)	151 (62.3%)	144 (59.8%)	0.677
Vasopressor, n (%)	136 (28.2%)	55 (22.8%)	81 (33.6%)	0.166
Clinical Outcomes				
LOS ICU, day	4 (1-12)	4 (2-12)	4 (1-13)	0.265
LOS Hospital, day	10 (5-20)	9 (5-18)	11 (4-22)	0.433
In-hospital mortality, n (%)	89 (18.5%)	29 (12.0%)	60 (24.9%)	< 0.001
ICU mortality, n (%)	65 (13.5%)	21 (8.7%)	44 (18.3%)	0.002
30-day mortality, n (%)	94 (19.5%)	33 (13.7%)	61 (25.3%)	0.001
90-day mortality, n (%)	121 (25.1%)	46 (19.1%)	75 (31.1%)	0.002
1-year mortality, n (%)	149 (30.9%)	60 (24.9%)	89 (36.9%)	< 0.001

**Figure 4:**
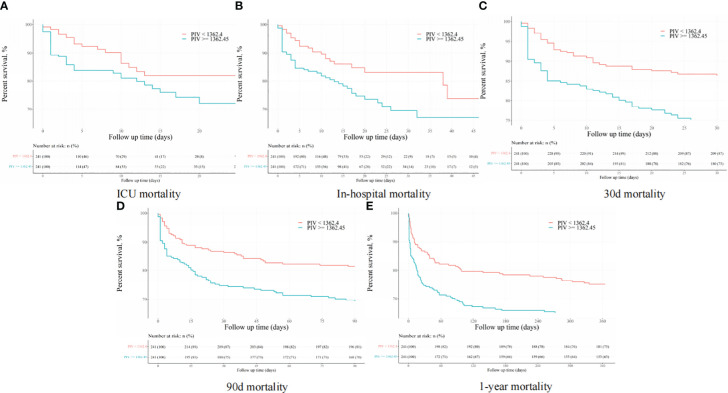
The K-M survival plots of **(A)** ICU, **(B)** in-hospital, **(C)** 30-day, **(D)** 90-day, and **(E)** 1-year mortality after propensity score matching.

After PSM, discernible disparities continued to exist between the two cohorts concerning 90-day (31.1 vs. 19.1%, P = 0.002), ICU (18.3 vs. 8.7%, P = 0.002), in-hospital (24.9 vs. 12.0%, P < 0.001), 30-day (25.3 vs. 13.7%, P = 0.001), and 1-year (36.9 vs. 24.9%, P < 0.001) all-cause mortality rates. Meanwhile, no significant variances were evident in length of stay (LOS) in the ICU (P = 0.265) and in the hospital (P = 0.433) (**Table 4**). Additionally, outcomes of the multivariate Cox regression analyses in patients following PSM manifested that a PIV ≥ 1362.45 retained its status as an independent prognosticator of increased mortality in the ICU (HR = 2.33, 95% CI: 1.11-4.91, P = 0.016), during hospitalization (HR = 2.06, 95% CI: 1.10-3.84, P = 0.034), at 30-day (HR = 1.66, 95% CI: 1.11-2.97, P = 0.047), 90-day (HR = 1.58, 95% CI: 1.14-2.67, P = 0.042), and 1-year (HR = 1.56, 95% CI: 1.10-2.53, P = 0.044) time points (**Table 5**). Moreover, K-M survival curves contrasting the two groups highlighted that even after PSM, patients with a PIV value ≥ 1362.45 consistently demonstrated significantly diminished survival rates at 90-day (68.9 vs. 80.9%, P < 0.001), within the ICU (81.7 vs. 91.3%, P < 0.001), during hospital stay (75.1 vs. 88.0%, P < 0.001) 30-day (74.7 vs. 86.3%, P < 0.001), and 1-year (63.1 vs. 75.1%, P < 0.001) intervals compared to patients with a PIV value < 1362.45 (**Figure 4**).

**Table 5:** Univariate and multivariate Cox regression models of PIV with mortality in patients with non-traumatic SAH after propensity score matching.

Outcome	Unadjusted		Model 1		Model 2	
	HR, 95% CI	P value	HR, 95% CI	P value	HR, 95% CI	P value
ICU mortality						
PIV (< 1362.45)	1 (Ref)		1 (Ref)		1 (Ref)	
PIV (≥ 1362.45)	1.90 (1.15-3.15)	0.013	1.92 (1.15-3.23)	0.014	2.33 (1.11-4.91)	0.016
In-hospital mortality						
PIV (< 1362.45)	1 (Ref)		1 (Ref)		1 (Ref)	
PIV (≥ 1362.45)	1.83 (1.18-2.82)	0.007	1.82 (1.17-2.85)	0.008	2.06 (1.10-3.84)	0.034
30-day mortality						
PIV (< 1362.45)	1 (Ref)		1 (Ref)		1 (Ref)	
PIV (≥ 1362.45)	1.92 (1.27-2.91)	0.002	1.96 (1.29-3.00)	0.002	1.66 (1.11-2.97)	0.047
90-day mortality						
PIV (< 1362.45)	1 (Ref)		1 (Ref)		1 (Ref)	
PIV (≥ 1362.45)	1.67 (1.17-2.39)	0.005	1.73 (1.20-2.50)	0.003	1.58 (1.14-2.67)	0.042
1-year mortality						
PIV (< 1362.45)	1 (Ref)		1 (Ref)		1 (Ref)	
PIV (≥ 1362.45)	1.62 (1.17-2.23)	0.004	1.71 (1.23-2.38)	0.002	1.56 (1.10-2.53)	0.044

Model 1: Unadjusted.

Model 2: Adjusted age, gender, and ethnicity.

Model 3: Adjusted all variables of P < 0.05 in **Table 2**.

### Subgroup analysis for the PIV on clinical outcomes in patients with non-traumatic SAH

**Figure 5:**
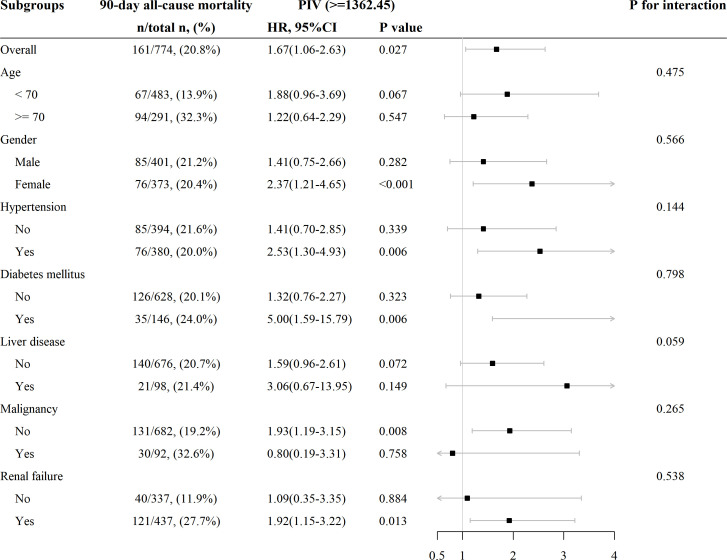
Subgroup analysis for the effect of PIV on 90-day all-cause mortality in patients with non-traumatic SAH.

Subgroup analyses were conducted to examine the association between SIRI and 90-day all-cause mortality in patients with AIS based on age (< 70 and ≥ 70 years), gender, hypertension, diabetes mellitus, liver disease, malignancy, and RF. The results revealed a consistent relationship between increasing PIV and higher 90-day all-cause mortality across all subgroups (**Figure 5**). All the stratification factors did not significantly affect the relationship between PIV and 90-day all-cause mortality.

### Regression cubic splines

**Figure 6:**
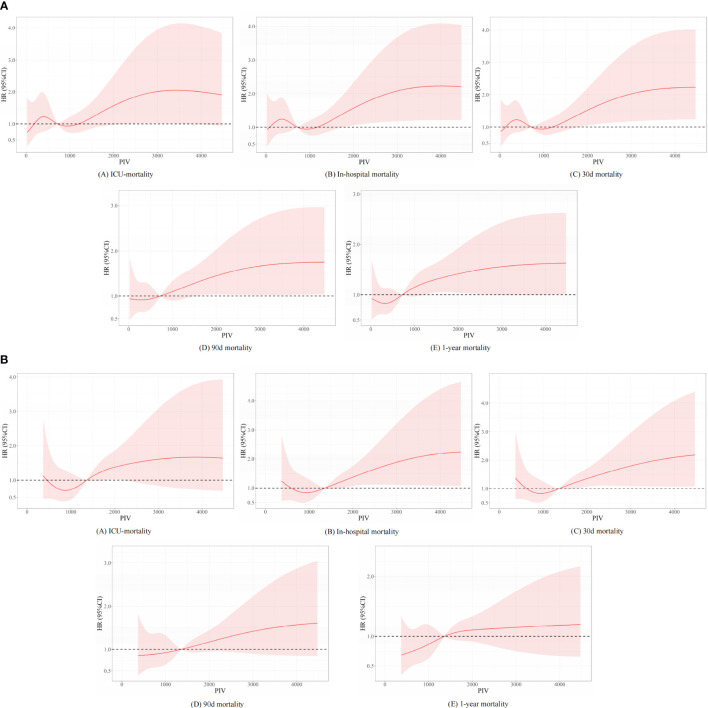
Underlying non-linear correlations between PIV and short- and long-term all-cause mortality **(A)** before and **(B)** after propensity score matching.

Besides, we further analyzed the original data when they were regarded as continuous variables. And we found that there are no statistical differences between different subgroups in the full-adjusted model 2 in short- and long-term all-cause mortality. Nevertheless, statistical differences between different subgroups were found in model 1 in terms of long-term all-cause mortality. In the unadjusted model, we found that statistical differences between different subgroups were found in terms of long-term all-cause mortality, except for ICU mortality. We described the related statistical results in **Supplemental Table 3** and **Figure 2**. To explore non-linear relationships, we employed restricted cubic splines (RCSs). Smooth curve fitting and generalized additive models were used to investigate the threshold effect of PIV on all-cause mortality in critically ill patients with non-traumatic SAH and identify the inflection point. A non-linear correlation was detectable between PIV and the propensity of ICU, in-hospital, 30-day, 90-day, and 1-year mortality before and after PSM, and the detailed statistical results can be found in **Figure 6**.

## Discussion

Given its broader cell type inclusion compared to other indices (SII or SIRI), PIV is perceived to be a superior indicator of inflammation, but it remains underutilized in the clinical sphere. The effectiveness of PIV has been tested in diverse scenarios, like being a predictive marker for small cell lung carcinoma (35), determining the risk of extended hospitalization post-elective thoracic surgery (36), and as an estimator for severity and ICU necessity in COVID-19 patients (37). Another piece of research has underscored the prognostic value of PIV in forecasting adverse outcomes for idiopathic pulmonary fibrosis patients (38). PIV quantifies inflammation by integrating various cell types participating in the immune response, such as neutrophils, lymphocytes, and platelets, along with monocyte counts. These cells are involved in the production of proinflammatory compounds like cytokines, chemokines, enzymes, and reactive oxidative species that fuel inflammation and instigate certain diseases (32). PIV, as a potential aggregate index, may assess the systemic inflammatory state and assess the balance between boosting and inhibiting inflammatory reactions. PIV is a simple biomarker that can be extracted from serum and is appropriate for long-term surveillance. Our study, for the first time, demonstrated that PIV plays a critical role in inflammation of non-traumatic SAH and is correlated with all-cause mortality.

It is becoming increasingly apparent that SAH is not solely a condition of the central nervous system. Rather, it has systemic implications, impacting not only the CNS, but also the cardiac and respiratory systems, as well as triggering systemic inflammatory responses (39–42). A growing body of research examining the interplay of inflammatory reactions and immune system dysregulation following non-traumatic SAH has indicated a significant involvement of the autonomic nervous system. Specifically, these studies point toward an upregulated sympathetic activity emanating from the hypothalamic-pituitary-adrenal axis (43). This heightened activity stimulates systemic inflammation, given that significant clusters of immune cells are responsive to catecholamines and cortisol (40, 43). Neutrophils, the preeminent leukocyte subtype in the human body, are crucial in mitigating both acute and chronic inflammatory reactions, undertaking phagocytic roles, and orchestrating the dissemination of anti-inflammatory agents (40, 44). On the other hand, lymphocytes have an essential part in both the instigation and resolution phases of inflammatory processes, with their functional state being either stimulated or inhibited based on diverse signaling pathways. The infiltration of lymphocytes contributes significantly to the initiation and escalation of inflammatory reactions, underlying the tissue deterioration and functional anomalies observed in inflammatory diseases (40, 41). Additionally, monocytes, another white blood cell variant, participate in the immune response and the onset of inflammation (40, 42). An escalated platelet to lymphocyte ratio (PLR) is deemed a negative prognostic indicator in the context of inflammatory disorders, given that an upsurged platelet count may lead to a reduction in lymphocyte count, a condition known as lymphopenia (43, 44). Hypothetically, neutrophils and lymphocytes could have a more pronounced influence on the pathogenesis of non-traumatic SAH compared to other cell types, and the assessment of multiple cellular categories in PIV evaluations might lead to an apparent dilution of their influence. While an elevated PIV proved beneficial in identifying patients at risk of mortality, the utilization of a solitary PIV measurement may not serve as an efficient instrument for gauging risk subsequent to a non-traumatic SAH. It is imperative to contemplate additional evaluation methodologies to corroborate the clinical significance of PIV within the non-traumatic SAH patient population.

The primary merit of our investigation lies in its reliance on extensive, real-world evidence, and the development of analogous cohorts via group matching, thus making strides toward diminishing bias resulting from confounding variables. Within the confines of our study, PIV exhibited commendable predictive prowess for mortality at any intervals. However, our study wasn’t without its limitations. First, we eliminated patients missing crucial data, such as WBC subtypes, potentially leading to a selection bias. Second, the study was confined to a single-center, and thus the predictive capacity of PIV for non-traumatic SAH warrants additional validation across diverse populations and geographical locales. Third, due to the retrospective data aggregation, variables were not uniformly disseminated among the groups, although PSM analysis was deployed to lessen disparities between cohorts. Fourth, given that the ICD code is a definitive diagnosis, immediate complications like stunned heart syndrome, pneumonia were excluded from our study, which may result in an inflated PIV due to severe exacerbation of cerebral perfusion and tissue necrosis. Hence, acknowledging these constraints is crucial when appraising the outcomes of this investigation, and future studies stand to gain by corroborating and augmenting these findings. Additional research is warranted to shed light on the underlying interplay between immune-related cellular entities and non-traumatic SAH patients, and to examine the potential shortcomings of utilizing systemic inflammatory response indices for inflammatory condition assessment.

## Conclusion

In critically ill patients suffering from non-traumatic SAH, an elevated PIV upon admission correlated with a rise in all-cause mortality at various stages, including ICU, in-hospital, the 30-day, 90-day, and 1-year mortality, solidifying its position as an independent mortality risk determinant. Nevertheless, to endorse the predictive value of PIV for prognosticating outcomes in non-traumatic SAH patients, additional prospective case-control studies are deemed necessary.

## Data availability statement

The original contributions presented in the study are included in the article/**Supplementary Material**. Further inquiries can be directed to the corresponding authors.

## Ethics statement

The studies involving humans were approved by The Institutional Review Boards of Massachusetts Institute of Technology (Cambridge, MA, USA) and Beth Israel Deaconess Medical Center (Boston, MA, USA). The studies were conducted in accordance with the local legislation and institutional requirements. Written informed consent for participation was not required from the participants or the participants’ legal guardians/next of kin because according to national legislation and institutional guidelines, written informed consent was not necessitated for this study.

## Author contributions

Y-WH conceptualized and planned the study. Y-WH and YZ were responsible for the initial drafting of the manuscript. Y-WH, YZ, and X-SY handled the collection and analysis of clinical data. Both Z-PL and Y-WH contributed to the statistical methodologies applied in the study and offered constructive suggestions. Z-PL and X-SY critically reviewed the study and partook in the interpretation of the results. All authors contributed to the article and approved the submitted version.
