# Supplementary material for: Association between a four-parameter inflammatory index and all-cause mortality in critical ill patients with non-traumatic subarachnoid hemorrhage: a retrospective analysis of the MIMIC-IV database (2012-2019)
Source: Front Immunol. 2023 Oct 23;14:1235266. doi: 10.3389/fimmu.2023.1235266 (PMC10626529; doi:10.3389/fimmu.2023.1235266)
Supplement: Supplementary file 1 [file DataSheet_1.pdf]

Supplemental Table 1. Missing values of included individuals.

Variable	Missing
Demographics	
Age	0
Men	0
Ethnicity	0
Clinical severity	
GCS	1
SAPS II	0
Vital signs	
SBP	1
DBP	1
MBP	1
Temperature	4
Heart rate	0
Respiratory rate	2
SpO_2	1
Comorbidities	
Hypertension	0
Diabetes mellitus	0

CHF	0
COPD	0
Sepsis	0
Vasopressor	0
Malignancy	0
Renal failure	0
Liver disease	0
Laboratory parameters	
WBC	18
Neutrophils percent	0
Lymphocytes percent	0
Monocytes percent	0
Platelets	21
Hemoglobin	18
Glucose	14
Chloride	14
Creatinine	14

BUN	14
PIV	0
Treatment	
Ventilation	0
Oxygen	0

Supplemental Figure 1. (A) the choice of optimal cut-off point that maximized the risk ratio and **(B)** the relationship between PIV ≥1362.45 and the distribution of PIV.

(A) **(B)**

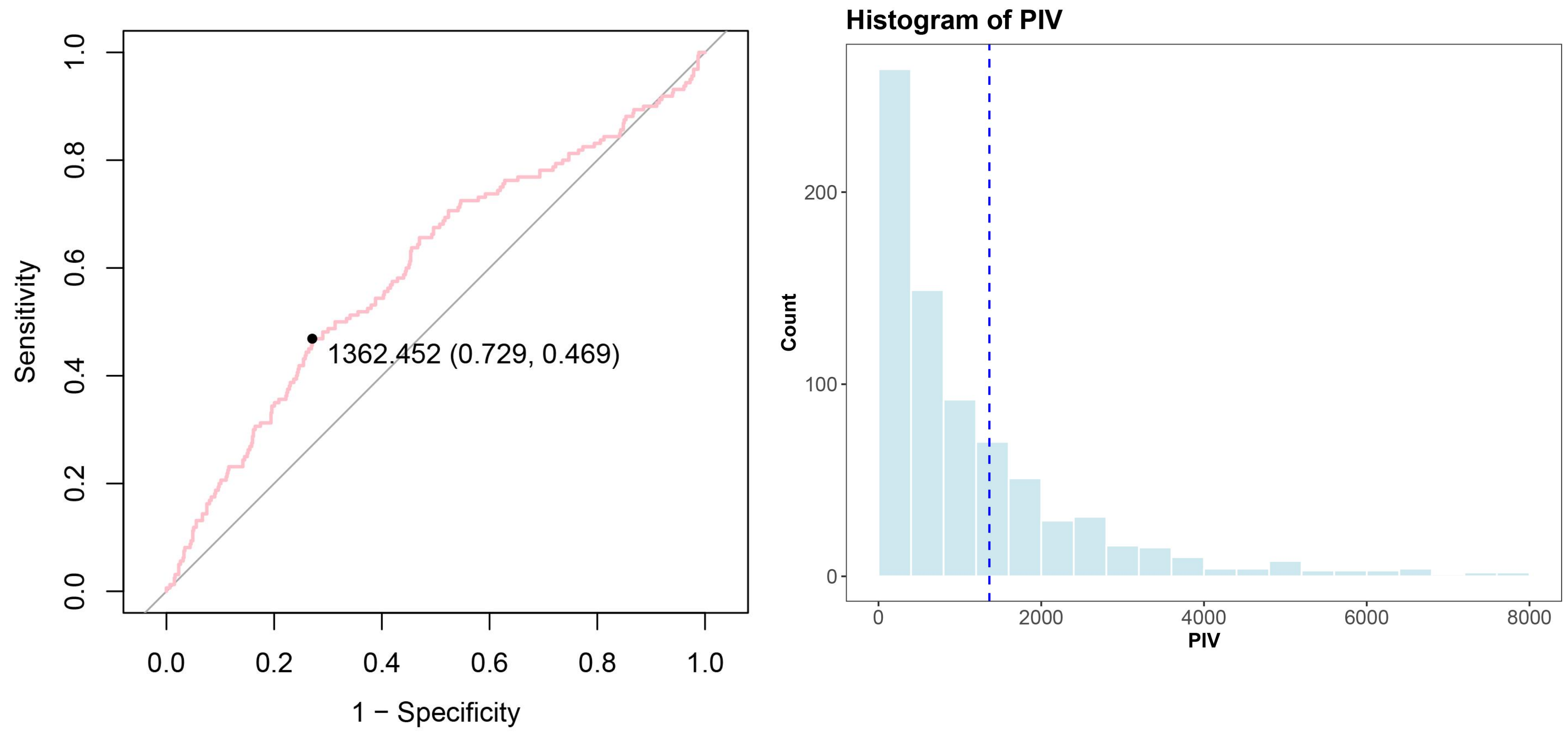

Supplemental Table 2. Baseline characteristics of included individuals and excluded individuals

Variable	Include (n=774)	Exclude (n=830)	*r* value
Demographics			
Age, years	62(51-76)	64(51-79)	0.112
Men, n (%)	401(51.8%)	394(47.5%)	0.082
Ethnicity, n (%)			<0.001
Asian	27(3.5%)	24(2.9%)	
White	450(58.1%)	514(61.9%)	
Black	45(5.8%)	41(4.9%)	
Others	252(32.6%)	251(30.2%)	
Clinical severity			
GCS	15(14-15)	15(15-15)	0.219
SAPS II	31(24-39)	32(23-40)	0.265
Vital signs			
SBP, mm Hg	132(115-146)	133(119-148)	0.126
DBP, mm Hg	72(62-83)	70(61-82)	0.511
MBP, mm Hg	88(78-100)	87(77-98)	0.200
Temperature	36.9(36.6-37.2)	36.8(36.3-37.1)	<0.001
Heart rate	82(71-94)	80(70-92)	0.022

Respiratory rate	18(15-21)	18(15-21)	0.120
SpO_2	99(96-100)	99(96-100)	0.433
Comorbidities			
Hypertension, n (%)	380(49.1%)	405(48.8%)	0.904
Diabetes mellitus, n (%)	146(18.9%)	128(15.4%)	0.067
CHF, n (%)	9(1.2%)	56(6.8%)	<0.001
COPD, n (%)	59(7.6%)	8(1.0%)	<0.001
Sepsis, n (%)	367(47.4%)	344(41.5%)	0.016
Vasopressor, n (%)	197(25.5%)	229(27.6%)	0.333
Malignancy, n (%)	92(11.9%)	104(12.5%)	0.694
Renal failure, n (%)	437(56.5%)	476(57.4%)	0.719
Liver disease n (%)	98(12.7%)	110(13.3%)	0.725
Laboratory parameters			
WBC, 10^9/L	11(8.3-14)	10.85(8.1-14)	0.693
Neutrophils percent, %	7.7(4.8-11.5)	.(.-.)	-

Lymphocytes percent, %	1.4(0.9-2.0)	.(.-.)	-
Monocytes percent, %	0.7(0.5-0.9)	.(.-.)	-
Platelets, $10^9/L$	195(159-240)	209(166-264)	<0.001
Hemoglobin	11.9(10.6-13.1)	12.1(10.7-13.4)	0.045
Glucose	127(106-151)	130(110-161)	0.015
Chloride	104(101-107)	105(102-108)	<0.001
Creatinine	0.8(0.7-1)	0.8(0.7-1)	0.304
BUN	14(10-20)	15(11-20)	0.142
PIV	722.4(292.8-1626.8)	.(.-.)	-
Treatment			
Ventilation, n (%)	531(68.6%)	622(74.9%)	0.005
Oxygen, n (%)	474(61.2%)	535(64.5%)	0.182

Supplemental Table 3. Univariate and multivariate Cox regression models used to study the association of PIV with mortality in patients with SAH (continuous variable).

Outcome	Unadjusted		Model 1		Model 2	
	HR (95% CI)	P value	HR (95% CI)	P value	HR (95% CI)	P value
**ICU mortality**						
< 292.80	Ref.	Ref.	Ref.	Ref.	Ref.	Ref.
292.80-722.44	0.78 (0.42-1.53)	0.497	0.74 (0.37-1.45)	0.379	1.25 (0.58-2.69)	0.564
722.44-1626.78	1.01 (0.58-1.84)	0.983	0.88 (0.46-1.67)	0.686	1.37 (0.62-3.03)	0.434
> 1626.78	1.46 (0.83-2.57)	0.189	1.06 (0.58-1.94)	0.840	2.01 (0.71-5.71)	0.189
P for trend		0.206		0.665		0.607
**In-hospital mortality**						
< 292.80	Ref.	Ref.	Ref.	Ref.	Ref.	Ref.
292.80-722.44	0.75 (0.42-1.33)	0.332	0.65 (0.36-1.18)	0.155	0.90 (0.46-1.74)	0.749
722.44-1626.78	0.94 (0.55-1.60)	0.829	0.74 (0.43-1.30)	0.298	0.95 (0.48-1.88)	0.889
> 1626.78	1.52 (0.95-2.44)	0.082	1.07 (0.64-1.77)	0.805	1.37 (0.56-3.33)	0.486
P for trend		0.032		0.200		0.596
**30d-mortality**						
< 292.80	Ref.	Ref.	Ref.	Ref.	Ref.	Ref.
292.80-722.44	0.83 (0.48-1.44)	0.512	0.75 (0.43-1.32)	0.324	0.92 (0.49-1.71)	0.779
722.44-1626.78	1.08 (0.64-1.81)	0.783	0.88 (0.51-1.51)	0.632	0.90 (0.47-1.71)	0.737
> 1626.78	1.90 (1.19-3.03)	0.007	1.43 (0.88-2.35)	0.153	1.28 (0.55-2.94)	0.568
P for trend		0.002		0.046		0.626
**90d-mortality**						
< 292.80	Ref.	Ref.	Ref.	Ref.	Ref.	Ref.
292.80-722.44	0.81 (0.49-1.35)	0.418	0.69 (0.41-1.17)	0.096	0.86 (0.49-1.52)	0.865
722.44-1626.78	1.34 (0.85-2.11)	0.210	1.19 (0.69-1.80)	0.645	1.19 (0.67-2.12)	0.560

> 1626.78	1.93 (1.26-2.98)	0.003	1.47 (0.93-2.32)	0.172	1.46 (0.68-3. 16)	0.336
P for trend		< 0.001		0.018		0.453
1-year mortality						
< 292.80	Ref.	Ref.	Ref.	Ref.	Ref.	Ref.
292.80-722.44	0.90 (0.57- 1.41)	0.644	0.78 (0.49- 1.24)	0.289	0.92 (0.56- 1.52)	0.738
722.44- 1626.78	1.45 (0.97-2. 19)	0.073	1. 19 (0.80- 1.82)	0.421	1.28 (0.75-2. 17)	0.364
> 1626.78	1.94 (1.31-2.88)	0.001	1.48 (0.98-2.24)	0.060	1.51 (0.74-3.05)	0.256
P for trend		< 0.001		0.018		0.233

Model 1: Unadjusted;

Model 2: Adjusted age, gender, and ethnicity;

Model 3: Adjusted all variables in Table 2 P < 0.05.

Supplemental Figure 2. The K-M survival plots of **(A)** ICU, **(B)** in-hospital, **(C)** 30-day, **(D)** 90-day, and **(E)** 1-year mortality (continuous variable).

(A)

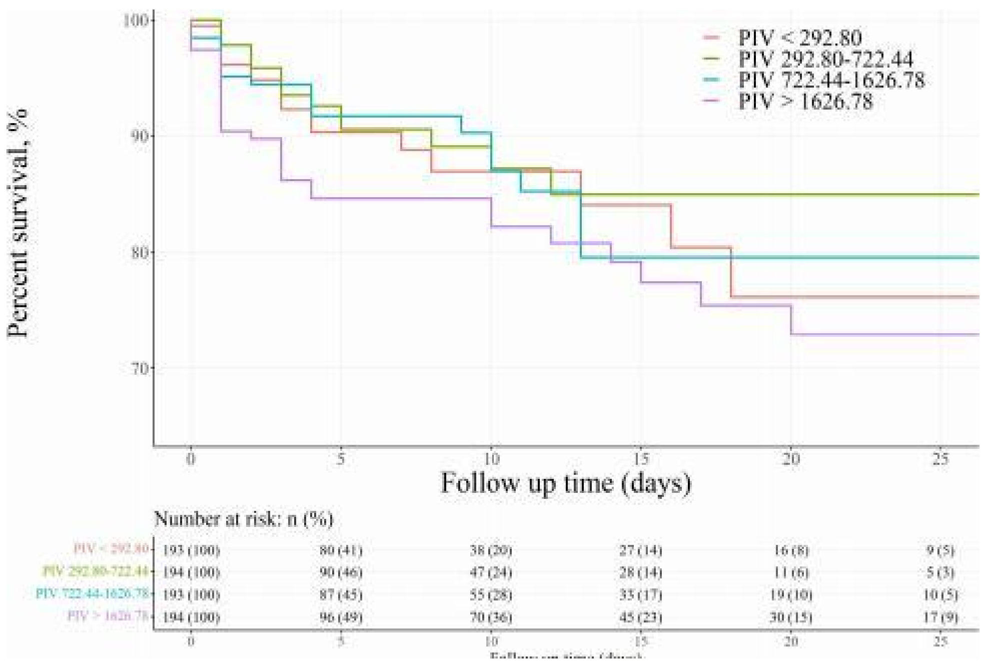

(B)

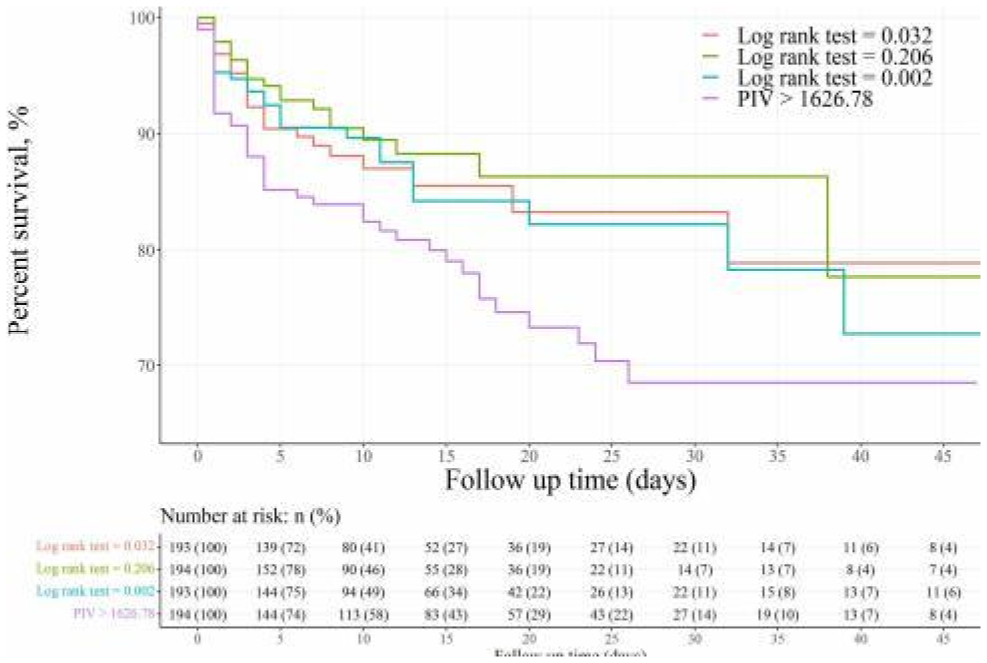

(C)

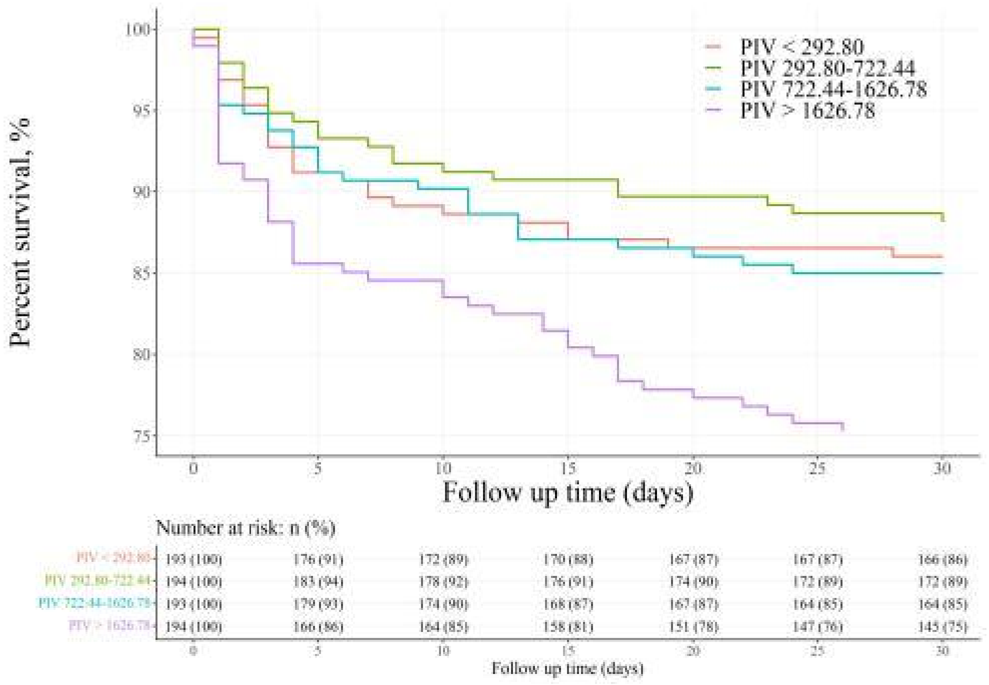
PIV < 292.80
PIV 292.80-722.44
PIV 722.44-1626.78
PIV > 1626.78
Percent survival, %
100
95
90
85
80
75
0
5
10
15
20
25
30
Follow up time (days)
Number at risk: n (%)
PIV < 292.80 193 (100) 176 (91) 172 (89) 170 (88) 167 (87) 167 (87) 166 (86)
PIV 292.80-722.44 194 (100) 183 (94) 178 (92) 176 (91) 174 (90) 172 (89) 172 (89)
PIV 722.44-1626.78 193 (100) 179 (93) 174 (90) 168 (87) 167 (87) 164 (85) 164 (85)
PIV > 1626.78 194 (100) 166 (86) 164 (85) 158 (81) 151 (78) 147 (76) 145 (75)
0
5
10
15
20
25
30
Follow up time (days)

(D)

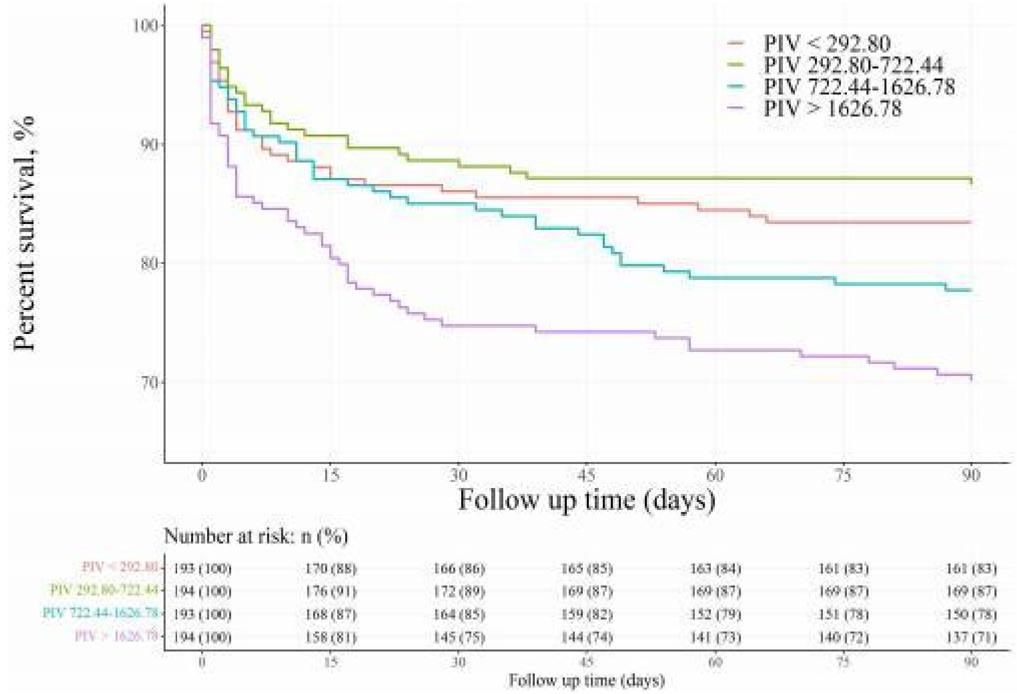
PIV < 292.80
PIV 292.80-722.44
PIV 722.44-1626.78
PIV > 1626.78
Percent survival, %
100
90
80
70
0
15
30
45
60
75
90
Follow up time (days)
Number at risk: n (%)
PIV < 292.80 193 (100) 170 (88) 166 (86) 165 (85) 163 (84) 161 (83) 161 (83)
PIV 292.80-722.44 194 (100) 176 (91) 172 (89) 169 (87) 169 (87) 169 (87) 169 (87)
PIV 722.44-1626.78 193 (100) 168 (87) 164 (85) 159 (82) 152 (79) 151 (78) 150 (78)
PIV > 1626.78 194 (100) 158 (81) 145 (75) 144 (74) 141 (73) 140 (72) 137 (71)
0
15
30
45
60
75
90
Follow up time (days)

(E)

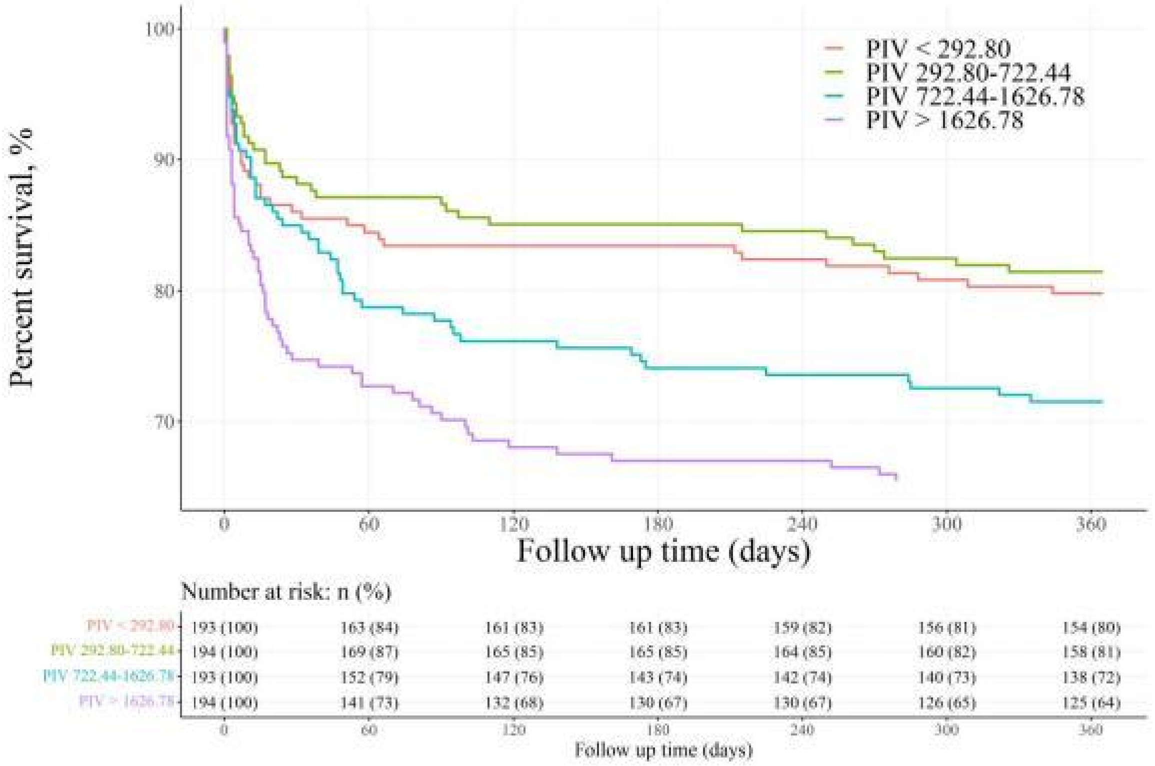
PIV < 292.80
PIV 292.80-722.44
PIV 722.44-1626.78
PIV > 1626.78
Percent survival, %
100
90
80
70
0
60
120
180
240
300
360
Follow up time (days)
Number at risk: n (%)
PIV < 292.80 193 (100) 163 (84) 161 (83) 161 (83) 159 (82) 156 (81) 154 (80)
PIV 292.80-722.44 194 (100) 169 (87) 165 (85) 165 (85) 164 (85) 160 (82) 158 (81)
PIV 722.44-1626.78 193 (100) 152 (79) 147 (76) 143 (74) 142 (74) 140 (73) 138 (72)
PIV > 1626.78 194 (100) 141 (73) 132 (68) 130 (67) 130 (67) 126 (65) 125 (64)
0
60
120
180
240
300
360
Follow up time (days)
